# Novel *β*-carbolines inhibit Wnt/*β*-catenin signaling

**DOI:** 10.1038/cddis.2015.335

**Published:** 2015-11-19

**Authors:** L Kong, B Mao, H Zhu, Y Li

**Affiliations:** 1 State Key Laboratory of Phytochemistry and Plant Resources in West China, Kunming Institute of Botany, Chinese Academy of Sciences, Kunming 650201, China; 2 State Key Laboratory of Genetic Resources and Evolution, Kunming Institute of Zoology, Chinese Academy of Sciences, Kunming 650201, China; 3 Chinese Center for Chirality, Key Laboratory of Medicinal Chemistry and Molecular Diagnostics of Education Committee of China, Hebei University, Baoding 071002, China

The evolutionarily conserved Wnt/*β*-catenin signaling pathway is involved in a multitude of developmental processes and the maintenance of adult tissue homeostasis, as well as by maintaining adult stem cells in a pluripotent state.^1^ Abberant activation of Wnt/*β*-catenin pathway was first linked to human cancer in the late 1990s, and was later found to contribute to development and progression of various cancers, especially colorectal cancer, mainly due to mutations in the genes encoding adenomatosis polyposis coli (APC), *β*-catenin and Axin.^2^ Though not yet in clinical application, Wnt signaling pathway has been a main target for antitumor drug development. In a recent issue of *Cell Death and Discovery*, we reported Isopropyl-ethyl-1-(naphthalen-1-yl)-9H-pyrido[3,4–b]-indole-3-carboxylate (Z86) as a novel Wnt/*β*-catenin signaling inhibitor with selective proliferation inhibitory activity on cancer cells *in vitro* and *in vivo*.^3^

In colorectal cancers where Wnt/*β*-catenin signaling is frequently activated by mutated APC or *β*-catenin, it seems that the ideal antagonist of the pathway would be the transcriptional complex of TCF and *β*-catenin in the nucleus. Accordingly, small molecules first identified as Wnt/*β*-catenin inhibitors targeted exactly the level of transcriptional complexes, such as NC043,^4^ Henryin^5^ and ICG001.^6^ However, there are recently some experimental results showing that, at least in some cases, targeting the upstream components of the Wnt signaling pathway can also have a role. The IWP compounds interfered the Wnt/*β*-catenin signaling through attenuating the production of Wnt ligands by targeting Porcupine, a member of the membrane-bound O-acyltransferase (MBOAT) family, which is essential for Wnt secretion and signaling ability.^7^ Recently, several small molecule inhibitors targeting the destruction complex composed of APC, Axin, GSK3*β* and other proteins of Wnt/*β*-catenin signaling were discovered. IWR,^8^ XAV939,^9^ JW55,^10^ J67 and J74^11^ promoted the phosphorylation and subsequent degradation of *β*-catenin by stabilizing of Axin, whereas the compound Pyrvinium^12^ enhances casein kinase to promote the phosphorylation of *β*-catenin and disturbs the stabilization of *β*-catenin.

In our study, from a chemical library consisting of 4000 chemically diverse compounds we firstly identified Z86 as a novel Wnt/*β*-catenin signaling inhibitor that belongs to *β*-carboline structure-type compound by using a cell-based luciferase reporter assay system.^3^ The inhibitory activities of the derivatives on Wnt/*β*-catenin signaling were investigated and the structure–activity relationship was characterized. The Wnt/*β*-catenin signaling inhibitory activity of Z86 was further confirmed in HEK293T cells transiently transfected with Wnt1, and cell lines with abberant activation of Wnt/*β*-catenin signaling, HCT116 and SW480 cells. Furthermore, Z86 inhibited the expression of endogenous Wnt/*β*-catenin signaling target genes and antagonized the second axis formation of Xenopus embryos induced by Wnt8. Further mechanism studies showed that Z86 treatment inhibits GSK3*β* (Ser9) phosphorylation, leading to its over-activation and promoted the phosphorylation and degradation of *β*-catenin (Figure 1). These results were supported by a recent report in which the authors identified a *β*-carboline alkaloid as a Wnt inhibitor, which seems to work similarly as Z86.^13^ Although we showed that reduced phosphorylation of GSK3*β* is involved in the inhibitory activity of Z86 on Wnt signaling, how Z86 activates GSK3*β* and inhibits *β*-catenin activity need further investigation. The identification of the targets and the disclosure of the mechanisms of Z86 will provide important basis for the development of a *β*-carboline alkaloid framework as anti-cancer agent targeting Wnt signaling pathway.

Constitutive Wnt/*β*-catenin signaling is essential for the colorectal cancer cell proliferation, and that the suppression of the Wnt/*β*-catenin signaling pathway can result in the inhibition of cell growth. Consistently, we further demonstrated that Z86 exhibited growth inhibitory effect on colorectal cancer cells through inducing G1 phase arrest of the cell cycle. Of note, there was no growth reduction observed in Z86-treated normal colonic epithelial cell line CCD-841-CoN cells, which are lack of aberrantly activated endogenous canonical Wnt signaling, indicating the selective growth inhibitory effect between cancer cells and normal cells was attributed to the inhibition of Wnt/*β*-catenin signaling. Notably, in nude mice models, Z86 dramatically inhibited the growth of tumors derived from xenografted HCT116 cells, which was associated with decreased GSK3*β* (Ser9) phosphorylation and increased *β*-catenin phosphorylation. Taken together, our findings provide a novel chemotype of antagonists of the canonical Wnt signaling and highlight a promising candidate for further colorectal cancer therapeutics development.

## Figures and Tables

**Figure 1:**
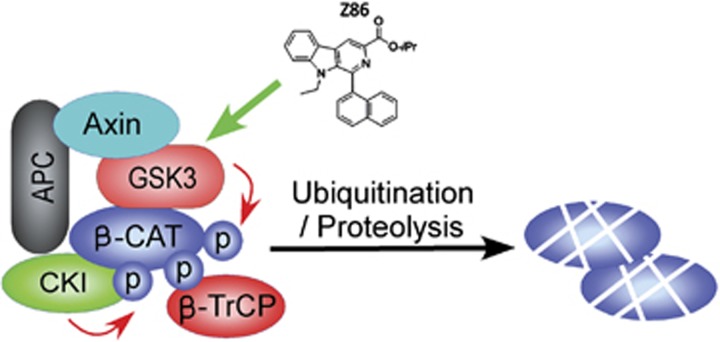
Z86 activated GSK3*β*, and promoted the phosphorylation of *β*-catenin and its subsequent degradation
